# Medical cannabis use among patients with Post-Traumatic Stress Disorder (PTSD): A nationwide database study

**DOI:** 10.1192/j.eurpsy.2023.306

**Published:** 2023-07-19

**Authors:** N. Yakirevich Amir, N. Treves, E. Davidson, O. Bonne, I. Matok

**Affiliations:** 1 Psychiatry, Hadassah University Medical Center; 2 School of Pharmacy, The Hebrew University of Jerusalem; 3 Anesthesiology, Hadassah University Medical Center, Jerusalem, Israel

## Abstract

**Introduction:**

In recent years, cannabis use among PTSD patients has become more common than ever. However, data available today regarding the effectiveness and safety of medical cannabis in PTSD treatment is limited, based on cross sectional studies, self-report surveys and a few clinical studies with small sample size.

**Objectives:**

To characterize patterns of use and adverse effects over time in patients with PTSD using medical cannabis in real life setting.

**Methods:**

Data were acquired from the Israeli national database of all patients licensed to use medical cannabis from January 2014 to December 2021. A license for medical cannabis is given to patients with PTSD of at least moderate intensity after treatment failure of at least two drugs and two psychological interventions. Comparative statistics were used to evaluate patterns of use and adverse effects.

**Results:**

12,977 patients were licensed to use medical cannabis in the study period for PTSD (8.2% of all users; 70% men) during the above-mentioned time period. PTSD was the 3^rd^ most common indication after chronic pain and symptoms related to oncological disease and chemotherapy treatment. Over time, the relative increase in use of medical cannabis among PTSD patients was higher than that found in non-PTSD patients. In 2021 36.2% of all PTSD patients using medical cannabis had their license issued that year compared to 28.1% of all non-PTSD patients. PTSD patients were significantly younger compared to non-PTSD patients (40.9 years vs. 52.9 years). PTSD patients consume slightly higher monthly amount at the beginning of treatment compared to non-PTSD patients (32.1gr vs. 30.6gr) with higher Tetrahydrocannabinol (THC) concentration (15.2% vs. 12.9%) and lower Cannabidiol (CBD) concentration (4.7% vs. 6.0%). Over two years of use, amount, and composition of cannabis among the two groups were comparable and showed an increase in total amount and THC concentration, reaching the maximal available THC concentration of 20%. Data regarding adverse effects were available for 6,242 PTSD patients (48.1%) and 39,497 non-PTSD patients (26.6%). PTSD patients reported more physical adverse effects (RR 1.45 [95%CI 1.34-1.56]), anxiety (RR 1.47 [95%CI 1.13-1.92]), and derealization (RR 3.44 [95%CI 2.42-4.89]).

**Conclusions:**

PTSD is one of the leading indications for medical cannabis use in Israel, despite scarcity in good quality data supporting its effectiveness and safety. The increased risk of mental adverse effects among PTSD patients emphasizes the need for cautious use in cannabis in this population. Expanding the knowledge regarding patterns of use and risks in medical cannabis use among PTSD patients is important for understanding the role of cannabis in PTSD treatment and to ensure an effective and safe treatment.

**Disclosure of Interest:**

None Declared

